# Anti-Inflammatory Properties of Dendrimers *per se*

**DOI:** 10.1100/tsw.2011.129

**Published:** 2011-07-07

**Authors:** Myriam Hayder, Séverine Fruchon, Jean-Jacques Fournié, Mary Poupot, Rémy Poupot

**Affiliations:** ^1^ Inserm, U1043, CNRS, U5282 and Université de Toulouse, UPS, Centre de Physiopathologie de Toulouse Purpan (CPTP), Toulouse, F-31300, France; ^2^ Inserm, U1037, CNRS, U5282 and Université de Toulouse, UPS, Centre de Recherche en Cancérologie de Toulouse (CRCT), Toulouse, F-31300, France

**Keywords:** dendrimers, inflammation, inflammatory disorders

## Abstract

Dendrimers are polybranched and polyfunctionalized tree-like polymers. Unlike linear polymers, they have perfectly defined structure and molecular weight, due to their iterative step-by-step synthesis. Their multivalent structure and supramolecular properties have made them attractive nanotools for applications, particularly in biology and medicine. Among the different biological and medical properties of dendrimers that have been developed over the past decades, the anti-inflammatory properties of several groups of dendrimers are the most recently discovered. Thereof, dendrimers emerge as promising, although heretical, drug candidates for the treatment of still-uncured chronic inflammatory disorders. This mini-review is based on the five main scientific articles giving an overview of what can be the spectrum of anti-inflammatory characteristics displayed by dendrimers.

## INTRODUCTION

### Short Historical and Semantic Preliminaries about Dendrimers

It is a generally acknowledged fact that the first report describing the synthesis of a series of "cascade molecules", compounds exhibiting potentially perpetual branching, was published by Buhleier et al. in 1978[1]. Earlier, the same group had described many-armed (although not branched) "octopus molecules"[2]. This term referred to both the structure of the molecules and their capability of extracting picric acid from a water solution. In the 1970s, "octopus" and "cascade" molecules where synthesized mainly as complex-forming ligands capable of solubilizing hydrophilic salts in aprotic organic solvents[3].

In the 1980s, after the proposal of "octopus" and "cascade" molecules, chemists vied with each other to find a name for this new family of molecules. "Tentacle molecules"[4] or "cauliflower polymers"[5] appeared. In 1985, Tomalia et al. referred to these radially symmetrical molecules as possessing "starburst" topology[6]. The word "dendrimer" appears for the first time in this report and, carefully reading the reference[7], it can be ascribed to A.J. Vogel as cited: "we acknowledge A.J. Vogel for coining this very appropriate term" in deference to their branched (Greek = dendritic; tree-like) as well as their oligomeric nature. "Dendrimer" was definitively popularized and widely established during the 1990s[8].

## What are Dendrimers?

Dendrimers belong to the polymer family. Unlike polydisperse linear polymers (molecules of different sizes), dendrimers are monodisperse polymers, i.e., their synthesis affords isomolecular species whose molecular size, shape, and disposition of organic moieties are perfectly controlled and adjusted. Dendrimers possess three distinguishable structural characteristics: (1) a central core unit, (2) one or several series of radial branches, and (3) functional groups that are affixed on the outermost series of branches. Dendrimers are built following either a divergent route (starting from the core unit) or a convergent route (starting from the functional groups)[9].

In divergent synthesis, the core unit is like a hub from which the radial growth of the dendrimer is initiated. After a first series of branches has been added onto the core, a point of divergence is created at the end of each branch. This point of divergence will enable the dendritic growth at the next step of the synthesis (Fig. 1). The branch and the divergence chemical pattern constitute the repeating unit of the dendrimer. The number of repeating units determines the generation of the dendrimer. Thus, starting with a core unit in the center, the branches can branch again and again, theoretically *ad libitum*. The divergent synthesis of dendrimers ends with the engraftment of functional groups at the points of divergence in the last generation of branches (Fig. 1). This iterative process of synthesis leads to a steric congestion of the numerous terminal groups at the periphery of the dendrimer. The core and repeating units make up the inner shell of the dendrimer, the functional groups constitute the outer shell at its periphery. Probably more than 50 different types of inner shells have been described so far. The molecular and electronic structure, and therefore the physicochemical properties (especially the hydrophilic/hydrophobic balance), of the inner shell account for specific interactions within the dendrimer, and thus affect both the global tridimensional scaffold of the dendrimer in its environment and its self-assembly properties[10].

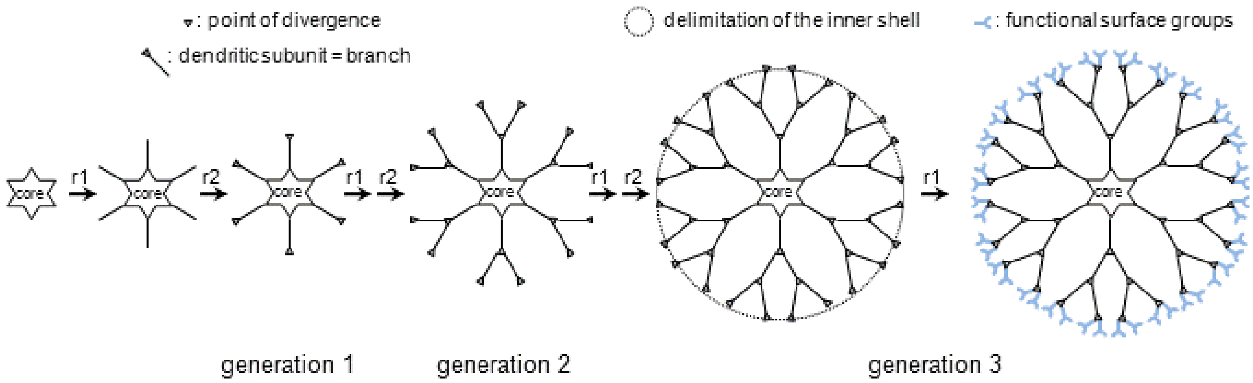

**FIGURE 1.** Scheme of the divergent synthesis route of a generation 3 dendrimer. r1 and r2 are the iterative reactions.

## Biomedical Applications of Dendrimers

Due to their supramolecular properties, dendrimers are attractive devices in a great variety of fields, such as described in Astruc et al.[11]: materials for optoelectronics and sensing (including biosensing), catalysis, imaging, or biological and medical applications. Of course, most of these fields largely overlap each other.

Very soon after the pioneering synthesis of dendrimers, this new family of molecules generated a great deal of attention for their use in biological and medical applications. Four main features of dendrimers underlie their successful emergence in the biomedical field:

1. Due to their sequential process of synthesis, dendrimers have perfectly defined structure and molecular weight. These are key points for the fate of dendrimers in biomedical applications, for the advent of new dendrimer-based therapeutics and diagnostic tools, in regard to regulation requirements.
2. Their supramolecular properties are strongly involved in their uses; i.e., supramolecular interactions with guest molecules inside the dendrimer and supramolecular interactions at the periphery of the dendrimer with substrates, molecular and/or cellular targets, or other dendrimers to generate nanodevices.
3. Their nanometric size and globular shape are comparable to those of biomolecules (such as nucleic acids and proteins) and supramolecular biostructures (such as biological membranes). One can assume that the size of a first-generation dendrimer begins at 1 or 2 nm and that, more or less, 1 nm in size is gained with each supplemental generation. Therefore, dendrimers undoubtedly pertain to the nanoworld. Together with their supramolecular properties, these structural characteristics make dendrimers perfect carriers of biomolecules and biomimics.
4. Their multivalency enables polyvalent interactions with biotargets. The majority of biological molecular interactions occur through polyvalent bindings[12]. The valency of a ligand corresponds to the number of separate cognate interactions of the same kind that can be established with its receptor(s). The strength of a single cognate interaction between a ligand and a receptor is called “affinity”. Natural ligands with multiple receptor binding sites (multivalent ligands) or multivalent engineered nanodevices interact through polyvalent interactions with their partner receptors. The strength of these polyvalent cognate interactions is called “avidity” (also “functional affinity”) and is much higher than the simple sum of the strengths of the single interactions. Thus, from monovalent to oligovalent and then polyvalent ligands, there is a strong enhancement in the intensity and duration of the stimulating signal that is delivered to a cell through a ligand-receptor interaction. From this point of view, dendrimers are perfect nanoplatforms to enable polyvalent interactions involving ligands that are originally monovalent and, thus, to alter a biological process[13]. Although the understanding of interactions between cells and nanostructures needs to be refined[14], appropriately designed dendrimers are potential therapeutics to activate a protective physiological response or to efficiently inhibit a deleterious pathological disorder.

Based on these underlying concepts, dendrimers burst onto the biomedical field[15] and are now part, in their own right, of the nanomedicine landscape[16]. Dendrimers can be designed for magnetic resonance imaging (MRI) as contrast agents, as well as for fluorescence imaging[17]. Recently, the design of a radiolabeled dendrimer for use in positron-emitted tomography (PET) as a nanoprobe specifically targeting $\alpha_v\beta_3$ integrin overexpressed in angiogenesis constitutes a paradigm of how the advantages of dendrimers can be combined to afford an innovative nanobiotool[18].

Biosensing techniques are another area that is innovatively using dendrimers. In particular, DNA microarrays and biosensors are a fast-developing business for dendrimers. Thanks to the need for genomic information in medicine (gene expression, mutation analyses), in forensic science (genotyping of individuals), and in analytical biochemistry (such as testing the safety and quality of food and environment), there is an increasing demand for more specific, more sensitive, and more user-friendly biosensors. Dendrimers are also involved in biosensors for antibodies or antigens, glucose, glutamate, and dopamine as diagnostic tools[11]. Due to the interactions that DNA and dendrimers display[19], the latter are also major transfection agents for gene or RNA delivery. Cationic dendrimers, via supramolecular electrostatic interactions with anionic nucleic acids, on the one hand, and negatively charged membrane surface on the other hand, bring the advantage of safety and versatility in comparison to viral vectors, especially regarding the intention of *in vivo* application (gene- and RNA-based therapies)[17,20].

As mentioned in the pioneering synthesis of dendrimers by Buhleier et al.[1], the rationale for the synthesis of large host molecules through a repetitive stepwise sequence of reactions was the inclusion of guest compounds in cavities or pseudocavities. Later on, nuclear magnetic resonance (NMR) studies of guest molecules in solution with starburst dendrimers of different generations suggested that these macromolecules (generation 4 and above) are able to encapsulate, and also aggregate at their surface, smaller guest molecules[21]. According to this study, encapsulation should be permitted when considering the predicted existence of void cavities in starburst dendrimers of the fourth generation and above. The first experimental evidence of a locked-in encapsulation of guest molecules in a dendritic structure designed and synthesized as such was afforded in 1994, with a diffusion of the guest out of the dendritic box, which was immeasurably slow[22]. The potential of dendrimers as drug nanocarriers has been recognized and explored since then. Different kinds of drugs have been encapsulated in or covalently conjugated with dendrimers. The objectives are to enhance the solubility of hydrophobic drugs in aqueous biological fluids[23]; to enhance the transdermal permeability of drugs, such as indomethacin (a nonsteroidal anti-inflammatory drug [NSAID])[24] or 5-fluorouracil[25]; to facilitate the intestinal absorption of poorly absorbable hydrophilic drugs and macromolecular compounds[26]; or to improve the pulmonary absorption of peptide and protein drugs[27]. The mechanisms by which a dendritic nanostructure can cross biological barriers are poorly depicted[28], but will benefit from studies regarding cellular responses mediated by nanoparticles[13].

The versatility of dendrimers enables the linkage of targeting functions at the surface of the nanocarrier, optimizing the biodistribution of the dendrimer-encapsulated or -conjugated drug. The specific targeting of the nanocarrier enables the site-specific delivery of the drug. Folic acid is the paradigm of the targeting group, which can be conjugated to dendrimers to target anticancer drugs to cancer cells[29]. In conclusion, the targeting of covalent and noncovalent drug-dendrimer nanoassemblies enables (1) the protection of the drug during its blood and tissue transit, (2) a lower dosage of the drug, (3) the avoidance of off-target effects of the drug, and, finally, (4) the controlled release of the drug to its target.

More recently, dendrimers *per se* also emerged as therapeutic agents. A wide variety of applications have been explored to promote these innovative drugs for prion diseases, neurodegenerative diseases such as Alzheimer's disease, viral and bacterial infections (including AIDS), tissue repair, cancer, and inflammatory diseases[30]. The most advanced dendrimer drug in clinical development ("VivaGel" from Starpharma) is intended for topical intravaginal use as an antiviral agent and is now in phase II clinical trials (http://clinicaltrials.gov/)[31]. In degenerative prion and Alzheimer's diseases, the beneficial effects of dendrimers are due to their direct interactions with detrimental accumulative peptide structures associated with these pathologies. Dendrimers can disrupt peptide aggregates and thereby block their deleterious accumulation. The direct anticancer properties of dendrimers that have been described are mediated by immunomodulation through interaction with cells of the immune system[32]. In this study, a glyco-conjugated dendrimer brings advantages on overall survival and tumor growth in a melanoma rat model by enhancing both the cytotoxicity of natural killer (NK) cells against the tumor and the activation of acquired immunity (CD4+ T lymphocytes).

The intrinsic anti-inflammatory properties of dendrimers are displayed mainly through immunomodulatory alterations of pathophysiological responses of the immune system. These properties have been proven *ex vivo* with human immune cells or *in vivo* in rodent models as reviewed below in a chronological order whenever logical.

## DENDRIMERS AND THE IMMUNE SYSTEM: ANTI-INFLAMMATORY PROPERTIES OF DENDRIMERS *PER SE*

### The Pioneering Work: Anti-Inflammatory Properties of Glyco-Conjugated PAMAM Dendrimers

The first report mentioning anti-inflammatory properties of dendrimers *per se* can be traced back to 2004[33]. Although the prevention of scar tissue formation is emphasized in its title, this article also presents the anti-inflammatory properties of a glucosamine-conjugated dendrimer towards human immune cells. The dendrimers that have been tested in this study are based on a 1,2-diaminoethane-cored generation 4.5 poly(amidoamine) (PAMAM) skeleton ended by 64 carboxylic acid groups, nine of which (14%) had been amido-conjugated to glucosamine (MW = 13.6 kDa) (Fig. 2) and glucosamine-6-sulfate (MW = 14.0 kDa).

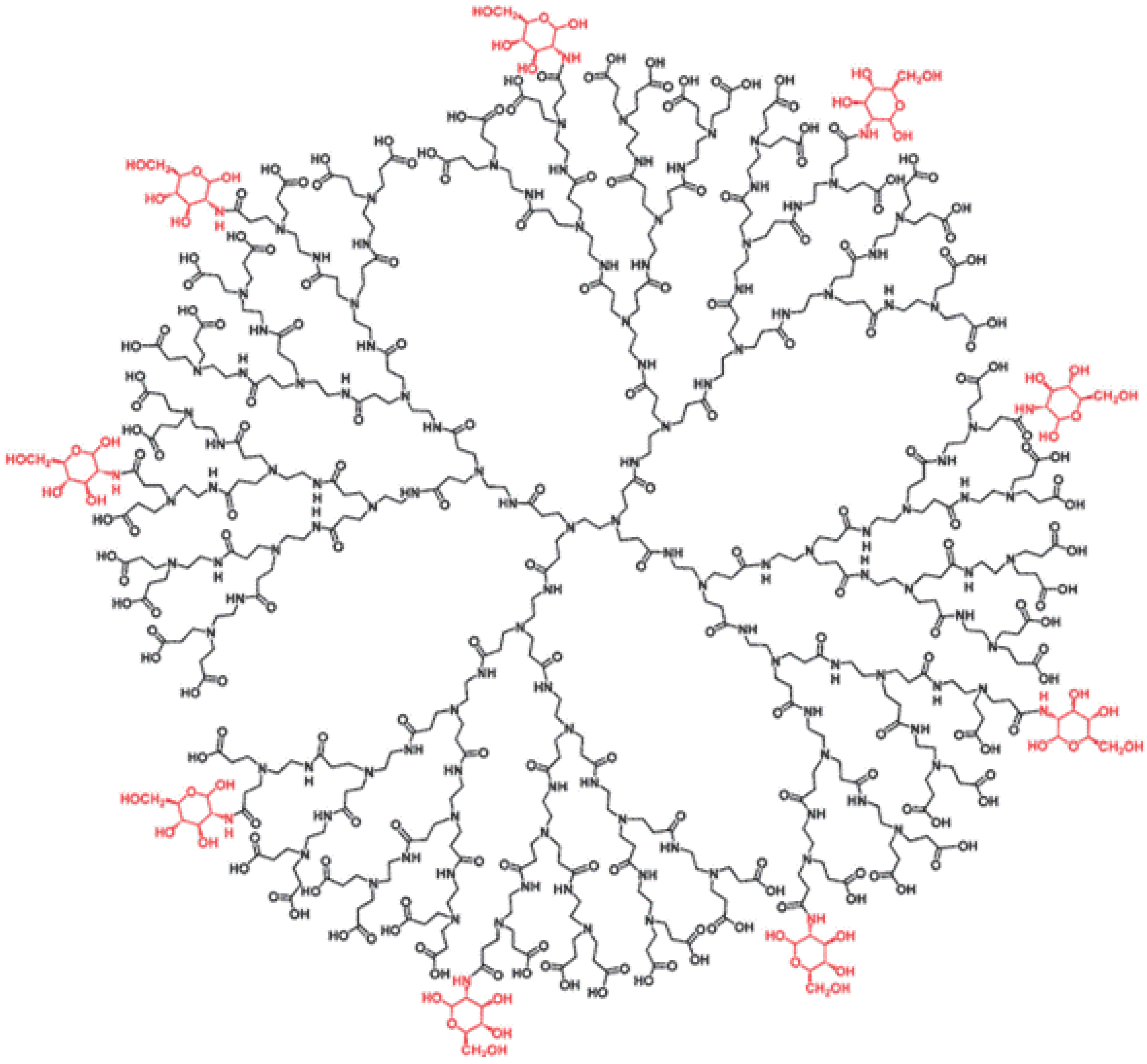

**FIGURE 2.** Structure of the glyco-conjugated dendrimer DG. In red, the amino-linked glucosamine surface groups. (From Shaunak et al.[33]. © Nature Publishing Group, reproduced with permission.)

The anti-inflammatory property of dendrimer glucosamine (DG) was evaluated by measuring the release of proinflammatory chemokines (macrophage inhibitory protein [MIP]-1α and -1β, interleukin [IL]-8) and cytokines (tumor necrosis factor [TNF]-α, IL-1β, and IL-6) by different immune cells stimulated during 21 h by *Salmonella minnesota* lipopolysaccharide (LPS). Immune cells were exposed to DG 30 min prior to LPS activation, or DG was added 2 or 4 h after the beginning of activation by LPS. In all these experimental settings, DG inhibited the LPS-mediated release of chemokines and cytokines by total peripheral blood mononuclear cells (PBMCs) with a 50% inhibitory concentration ($IC_{50}$) of 6.8 ± 1.1 µM. These experiments have been repeated on purified populations of cells, demonstrating that the primary effect of DG is on monocyte-derived macrophages (MDMs) and immature monocyte-derived dendritic cells (DCs). The inhibition of chemokine and cytokine release has been confirmed at the mRNA level by quantitative real-time polymerase chain reaction (PCR). Moreover, LPS stimulation induced at various time intervals after DG exposure shows that the anti-inflammatory effect of DG is reversible.

An immunosuppressive effect of DG is also demonstrated in this study, insofar as this dendrimer inhibits the proliferation of lymphocytes in mixed leukocyte reactions (MLR) between DCs and peripheral blood lymphocytes (PBLs) with an $IC_{50}$ of 5.1 ± 0.8 µM.

The second dendrimer used in this study is dendrimer glucosamine-6-sulfate (DGS). DGS harbors antiangiogenic activity proven by the *in vitro* inhibition of the proliferation of human umbilical vein endothelial cells (HUVECs).

Also, the toxicity of DG and DGS for a T-cell line and a macrophage cell line has been evaluated. With DG, the 50% lethal doses ($LD_{50}$) for the T-cell line and the macrophage cell line are, respectively, 134 ± 17 and 209 ± 8 µM. With DGS, $LD_{50}$ are 22 ± 2 and 19 ± 1 µM, respectively. When added at 15 µM (DG) and 7 µM (DGS) in culture of PBMCs, MDMs, DCs, T lymphocytes, or HUVECs, no adverse effect on cell viability or growth is observed.

The combination of the immunomodulatory dendrimer DG and the antiangiogenic dendrimer DGS has been tested *in vivo* onto the subconjunctival scarring in a rabbit model after glaucoma filtration surgery. Postsurgical scarring is due to a persistent inflammatory and angiogenic response. The combination of dendrimers was administered in 15 injections (beginning at day -2 before glaucoma surgery and ending at day 28 after it). The total amounts of DG and DGS were, respectively, 60.30 and 30.15 mg, 99% of which were injected by the intraperitoneal route, the remaining 1% was injected by the subconjunctival route. The efficacy of the treatment was inferred by the persistence of bleb after surgery, indicating an excessive scar tissue formation. This experiment shows a dramatic effect of the combination of DG and DGS, increasing the long-term success of surgery from 30 to 80%.

## Anti-Inflammatory Properties of Phosphorus-Containing Dendrimers

In 2006, we reported that phosphorus-based dendrimers capped by amino-bisphosphonate groups, and especially dendrimer azabisphosphonate (ABP) presented in Fig. 3, have activating properties towards human monocytes[34]. These properties were depicted mainly as changes in the morphology and the phenotype of monocytes, increase of their phagocytosis activity, and their survival in culture at concentrations in the micromolar range (2 and 20 µM).

Here, we give away for the first time the rationale for the design of dendrimers ended by phosphorus-containing functions. It relies on the structural features of particular nonpeptide antigens that specifically activate a subpopulation of peripheral blood T cells, the so-called Vγ9Vδ2 T lymphocytes[35]. These cells are stimulated by small pyrophosphorylated molecules and have an antitumor cytotoxic activity that makes them potential effectors in cellular anticancer therapies[36]. We have shown that the pyrophosphate group is crucial for the bioactivity of these molecules[37] and proposed to call them phosphoantigens. In line with the concept that polyvalent ligands should enable higher functional affinity and finally stronger activation of target cells[12], as already evoked in this review, we proposed to the neighboring Majoral-Caminade research team to prepare a dendritic device bearing pyrophosphate groups at its surface as a potent activator of Vγ9Vδ2 T lymphocytes. Due to the instability of pyrophosphate

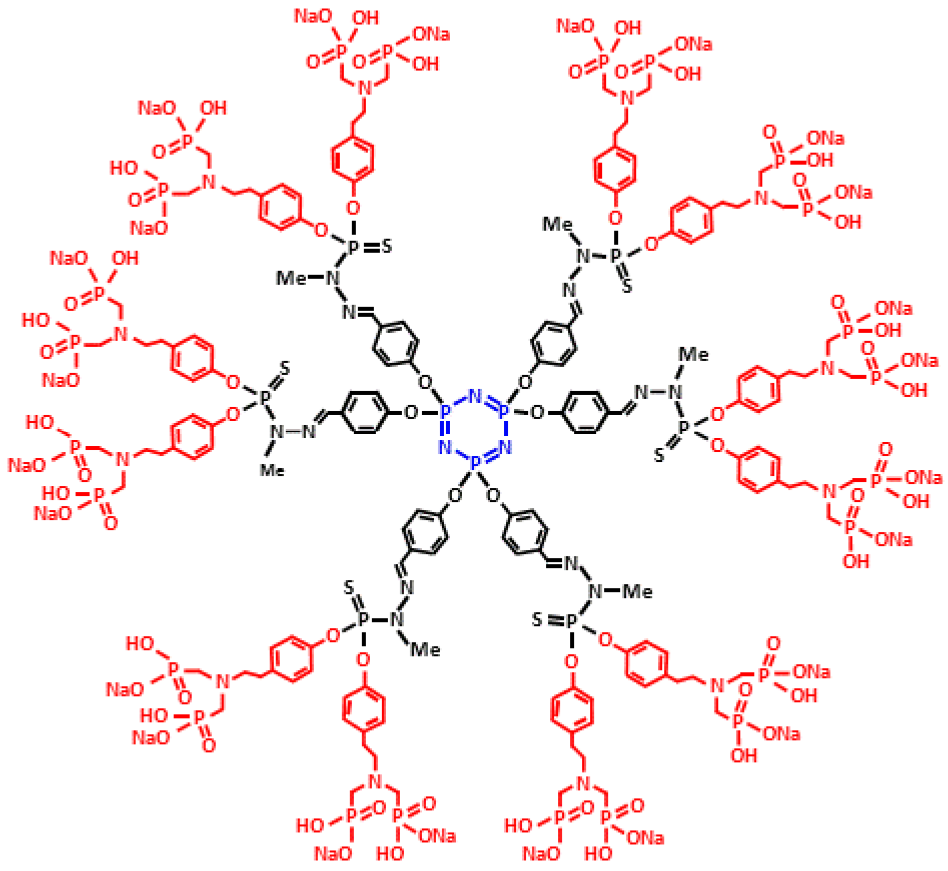

**FIGURE 3.** Structure of the phosphorus-containing dendrimer ABP used in Fruchon et al.[46]. In blue, the cyclo-triphosphazene ($N_3P_3$) core; in black, the phenoxymethyl-methylhydrazone branches; in red, the tyramine-based ABP surface groups.

in acidic environment, which makes the prospected synthesis hazardous, the first dendrimers we tested bore azamono- or azabisphosphonate groups instead of the phosphate-intended groups[38,39,40]. Phosphonate-capped dendrimers have a poor effect on the activation of Vγ9Vδ2 T lymphocytes, but twists and turns of research led us to the discovery of the unprecedented immunomodulatory effects of dendrimer ABP on the human immune system[41,42]. Aside from its effect on human monocytes, we found that dendrimer ABP promotes the amplification of human NK cells in cultures of PBMCs[43]. One of the cellular events leading to the proliferation of NK cells is the specific inhibition of the proliferation of CD4+ T lymphocytes by dendrimer ABP[44]. NK cells are cytotoxic effectors against virus-, bacteria-, or parasite-infected cells and against tumor cells. Therefore, NK cells are of particular interest for immunocellular therapies, especially for cancer treatments, provided their production in batches compliant with their use in human therapy from a quantitative (and qualitative) point of view. Dendrimer ABP is the first chemical compound proposed for the *ex vivo* production of NK cells starting with PBMCs from healthy donors or from cancer patients[45].

At the beginning of 2009, we published new results refining the activating properties of dendrimer ABP towards human monocytes[46]. We chose an overall, comprehensive approach comparing the transcriptional profiles of nonactivated and dendrimer-activated (*da*) human monocytes purified from three healthy donors. Monocytes had been activated for 6 h before preparing mRNA. We performed a high-standard statistical analysis of the results as genes were considered differentially regulated in *da* monocytes in comparison with nonactivated monocytes, if they had a fold change of ≥2.0 or ≤2.0 for at least two donors of the three. With these settings, 78 genes were found overexpressed and 62 genes were found underexpressed by *da* monocytes. Twenty-five of the up-regulated genes and 17 of the down-regulated genes were relevant of an anti-inflammatory activation of monocytes (also called alternative activation), displaying features of IL-4, IL-10, or IL-13 activation. This alternative-like activation of human monocytes by dendrimer ABP was confirmed by quantitative real-time PCR of four gene products characterizing the classical inflammatory activation of monocytes (one proinflammatory chemokine [CCL5] and three proinflammatory cytokines [IL-1β, IL-6, and IL-12]) and five gene products characterizing the alternative, anti-inflammatory activation of monocytes (the mannose receptor MRC1, IL-1RN, IL-10, CCL18, and CD23). The comparison of the level of gene transcripts in *da* monocytes and in nonactivated monocytes gave clear-cut results. The anti-inflammatory mRNA were significantly overexpressed in *da* monocytes, whereas the inflammatory RNA were either underexpressed or remained unmodified. Flow cytometry analyses at the protein level (i.e., the phenotype) of *da* monocytes, inflammatory monocytes, and anti-inflammatory monocytes showed that the expression of CD206 (mannose receptor MRC1) was strong in anti-inflammatory and *da* monocytes (contrary to inflammatory monocytes), whereas the expression of CD64 (Fcγ-RI) and CD13 (membranous aminopeptidase N) was decreased in anti-inflammatory and *da* monocytes in comparison with inflammatory monocytes.

The stimulatory properties of the three types of activated monocytes have been evaluated in MLRs. MLRs were assessed as the proliferation of CD4+ T lymphocytes triggered by the differently activated monocyte populations. These functional experiments also confirmed the close likeness of anti-inflammatory and *da* monocytes: both cells gave the weaker MLRs in comparison with inflammatory monocytes. What is more, the CD4+ T lymphocytes generated in the weak MLRs with anti-inflammatory and *da* monocytes are potent immunomodulatory cells as they produce IL-10.

Thus, dendrimer ABP has anti-inflammatory and immunomodulatory properties, either exerted directly towards monocytes or as the consequence of the activation of the latter on other immune cells. By some aspects, its *in vitro* properties match those of glucocorticoids, the most widely used immunosuppressive drugs[47]. Although the effect of dendrimer ABP remains to be challenged in *in vivo* models of inflammation, phosphorus-containing dendrimers may represent a new family of immunologically active drugs for the resolution of inflammatory disorders.

## Anti-Inflammatory Properties of PAMAM Nanocarriers Alone

Later in 2009, the unprecedented anti-inflammatory activity of simple surface-functionalized PAMAM dendrimers was revealed[48]. The prior objective of this work was the pharmacokinetic study on the well-known PAMAM dendrimers conjugated with indomethacin, an NSAID. In this study, naked dendritic nanocarriers were probably intended to be negative controls of the properties of PAMAM–indomethacin complexes. Three different rat models of inflammation were screened: (1) the acute model of carrageenan-induced paw edema, (2) the subacute cotton pellet model, and (3) the chronic model of adjuvant-induced arthritis. The dendrimers tested are based mainly on a 1,2-diaminoethane-cored generation 4.0/4.5 PAMAM skeleton ended by $–NH_2$ (G4-$NH_2$), –OH (G4-OH), and –COOH (G4.5-$CO_2H$) groups (Fig. 4). The latter corresponds to the dendrimer that had been derived in glucosamine-conjugated dendrimers, as seen in Shaunak et al.[33].

In the acute carrageenan-induced paw edema model, a single dose of test formulations was injected into the intraperitoneal cavity, just before the injection of the carrageenan solution in a paw. Edema was monitored for 8 h by measuring the volume of the injected paw. A first experiment compared the effect of dendrimer G4-$NH_2$ alone (8 mg/kg), indomethacin alone (1.6 mg/kg), and a complex of dendrimer G4-$NH_2$ and indomethacin (8 and 1.6 mg/kg, respectively). At all time intervals, the mean percentage of inhibition of the paw swelling was higher with the G4-$NH_2$–indomethacin complex in comparison with indomethacin alone, and G4-$NH_2$ alone exhibited the lowest effect. Nevertheless, 1 h after the inflammation had been induced, G4-$NH_2$ inhibited the paw swelling by 30%. This rate can be increased at around 45% with a dose of 16 mg/kg of G4-$NH_2$. Then, on the same test, the effects of G4-$NH_2$, G4-OH,

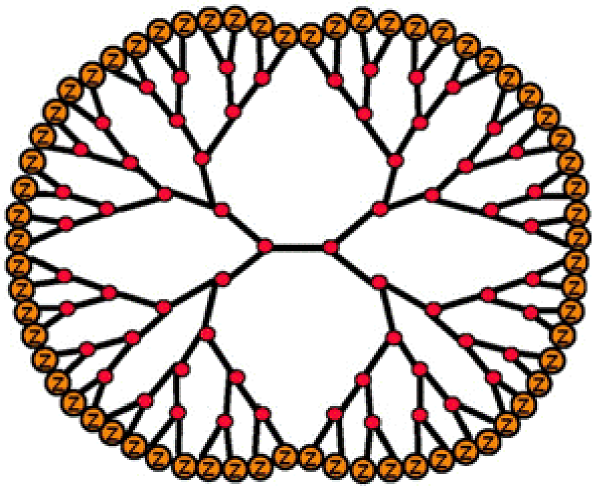

**FIGURE 4.** Schematic representation of 1,2-diaminoethane-cored generation 4.0 PAMAM dendrimers (if Z = $–NH_2$: G4-$NH_2$, if Z = –OH: G4-OH). (Reprinted with permission from Chauhan et al. *Biomacromolecules* **10**, 1195–1202. Copyright 2009 American Chemical Society.)

and G4.5-$CO_2$H were compared. The effects of G4-$NH_2$ and G4-OH seemed to be more or less the same, and G4.5-$CO_2$H exhibited substantially less activity, but the figure is missing in the article.

In the subacute cotton pellet test, G4-$NH_2$, indomethacin alone, and the G4-$NH_2$–indomethacin complex were compared. Test formulations were injected intraperitoneally daily from day 1 to 7. At day 8, rats were euthanized, and pellets with granuloma tissue were dried and weighed. Contrary to the carrageenan-induced paw edema model, the G4-$NH_2$–indomethacin complex and G4-$NH_2$ exhibited significantly higher mean percentage of inhibition of granuloma formation than indomethacin alone (47 and 50% vs. 22%).

With the third model, a preventive assay was performed insofar as daily intraperitoneal dosing of test formulations had been initiated at day -1 (prior to Freund's adjuvant injection) and until day +14. The progression of arthritis was assessed by measuring paw swelling at different days. In the early days, the inhibitory effect of G4-$NH_2$–indomethacin was higher than the effect of G4-$NH_2$ and indomethacin alone, in respective rank. Later on, the effects of G4-$NH_2$–indomethacin and G4-$NH_2$ were more or less the same and were significantly higher than that of indomethacin alone.

To gain deeper cellular and molecular insights into the effect and mechanism of the *in vivo* anti-inflammatory properties of these PAMAM dendrimers, authors investigated their effects on proinflammatory mediators such as nitric oxide (NO) and cyclo-oxygenases (COX) *in vitro*. The effect of PAMAM dendrimers was evaluated via the production of NO by rat peritoneal macrophages triggered by LPS. In a concentration range between 0.005 and 1 nM, G4-$NH_2$ and G4-OH exhibited slightly greater inhibitory activity compared to G4.5-$CO_2$H, but without any dose effect.

COX enzymes, and especially the inducible COX-2, are activated in an inflammatory context. Therefore, COX-2 inhibition is an accurate target for the development of anti-inflammatory drugs (NSAIDs). The authors ended their study with the screening of the effects of different dendrimers (generation, terminations) towards COX-2 *in vitro*. In a first series of experiments, dendrimers at the common generation level (i.e., G = 4.0) were tested at 0.174 w/v (more or less $10^{-4}$ M, depending on molecular weights). Amine- and hydroxyl-containing surface functions (G4-$NH_2$, the supplementary aminoethylethanolamine-capped dendrimer [G4-AEEA], and G4-OH) displayed the highest inhibitory activity of COX-2, between 53.7 ± 8.4 and 34.9 ± 4.9%, respectively. Other supplementary dendrimers ended by tris(hydroxymethyl)aminomethane (G4-Tris), N-(3-carbomethoxy) pyrrolidone (G4-Pyr), or polyethylene glycol (G4-PEG) groups showed decreasing inhibitory effects. Carboxylate-capped dendrimers (G4-$CO_2H$ and the supplementary succinamic acid–capped dendrimer [G4-SUC]) had no detectable effect on COX-2 inhibition. In a second series of tests, the effect of dendrimer generation was explored with AEEA-terminated dendrimers of generation 4.0, 5.0, and 6.0 at a concentration of 24.36 µM. Whereas G4-AEEA had no effect at this concentration on COX-2 inhibition, G5-AEEA and G6-AEEA dendrimers showed activity up to 42.5 ± 5.4% for the latter. This is the generation-dependent dendritic effect. However, no core effect had been noted in this study when comparing 1,2-diaminoethane– and 1,12-diaminododecane–cored dendrimers of the G5-AEEA and G6-AEEA series.

All in all, this article reports the various effects of different series of PAMAM dendrimers in *in vivo* and *in vitro* tests. Although it is difficult at this stage to delineate clear-cut structure-activity relationships — for instance, some dendrimers are active *in vivo,* but not *in vitro* — the inhibitory effect of some of these dendrimers on COX-2 is relevant in the current competition for the discovery of safe COX-2 inhibitors. This is of particular importance in fighting against cancers as prostanglandin $E_2$ ($PGE_2$), a final metabolite of the COX pathway, has strong immunosuppressive properties towards Vγ9Vδ2 T lymphocytes and NK cells, two major subsets of the immune system with antitumor cell cytotoxicty[49,50].

## Anti-Inflammatory Properties of Polyethylene Oxide (PEO) Dendrimers

So far, we have reviewed the anti-inflammatory properties displayed by dendrimers, focused towards immune cells of the myeloid lineage such as MDMs and immature DCs[33], peripheral blood monocytes[46], and peritoneal macrophages[48]. Nevertheless, one crucial step of the inflammatory response is the recruitment of the inflammatory effectors, or their circulating precursors, from the blood to the site of inflammation. Therefore, another potent form of anti-inflammatory therapy is to target this leukocyte trafficking[51]. Extravasation of leukocytes through the endothelial barrier to the sites of inflammation is initiated by selectin-induced leukocyte tethering and rolling on the endothelial surface. Selectins are glycoproteins of the lectin family, expressed both by leukocytes (L-selectin or CD62L) and endothelial cells (E- and P-selectins or CD62E and CD62P). In return, leukocytes express CD162 (or P-selectin glycoprotein ligand-1, PSGL-1), a high-affinity ligand of E- and P-selectins, whereas endothelial cells express the ligands of L-selectin: CD34 (or sialomucin) and glycosylation-dependent cell adhesion molecule-1 (Gly-CAM-1). These ligands are O-glycosylated proteins that present carbohydrate epitopes consisting of sulfated derivatives of the tetrasaccharide sialyl Lewis X motif. Generating sulfated glyco-conjugate analogs of sialyl Lewis X as antagonist ligands for selectins is a promising track in order to develop anti-inflammatory drugs.

In this aim, Rele et al.[52] synthesized three- and four-arm PEO (or polyethylene glycol, PEG) “stars” and a second-generation PEO dendrimer built on a $N_3P_3$ core (Fig. 5). These molecules were ended by lactose groups on which hydroxyls can be naked, acetylated, or sulfated. Their anti-inflammatory properties were compared to that of heparin (a sulfated polysaccharide), which exhibits anti-inflammatory properties by blocking L- and P-selectins via sulfate-dependent interactions. An acute inflammatory response was induced in mice by thioglycollate injection into the peritoneal cavity. Five minutes later, mice received intravenous injection of heparin or “star” and dendrimer analogs at 0.5 mg/mouse (i.e., 20 mg/kg). The recruitment of neutrophils and macrophages was quantified 3 h later in the peritoneal cavity. Whereas the three- and four-arm sulfated PEO “stars” showed little activity, the sulfated PEO dendrimer dramatically reduced the recruitment of neutrophils (86%, the same rate as heparin) and macrophages (60%, less than the heparin control). Once more, the dendritic scaffold takes advantage of a multivalent ligand presentation to have a similar degree of bioactivity than the natural polymer. As the effect of the sulfated PEO dendrimer was presumed to be mediated by a selectin-dependent blockade, the authors confirmed this assessment by an inhibition test of the adhesion of U937 lymphoma cells to immobilized E-, L-, or P-selectins *in vitro*. Heparin and the sulfated PEO dendrimer were unable to inhibit cell adhesion to E-selectin. These data were expected as E-selectin is the only one that has no positively charged motifs

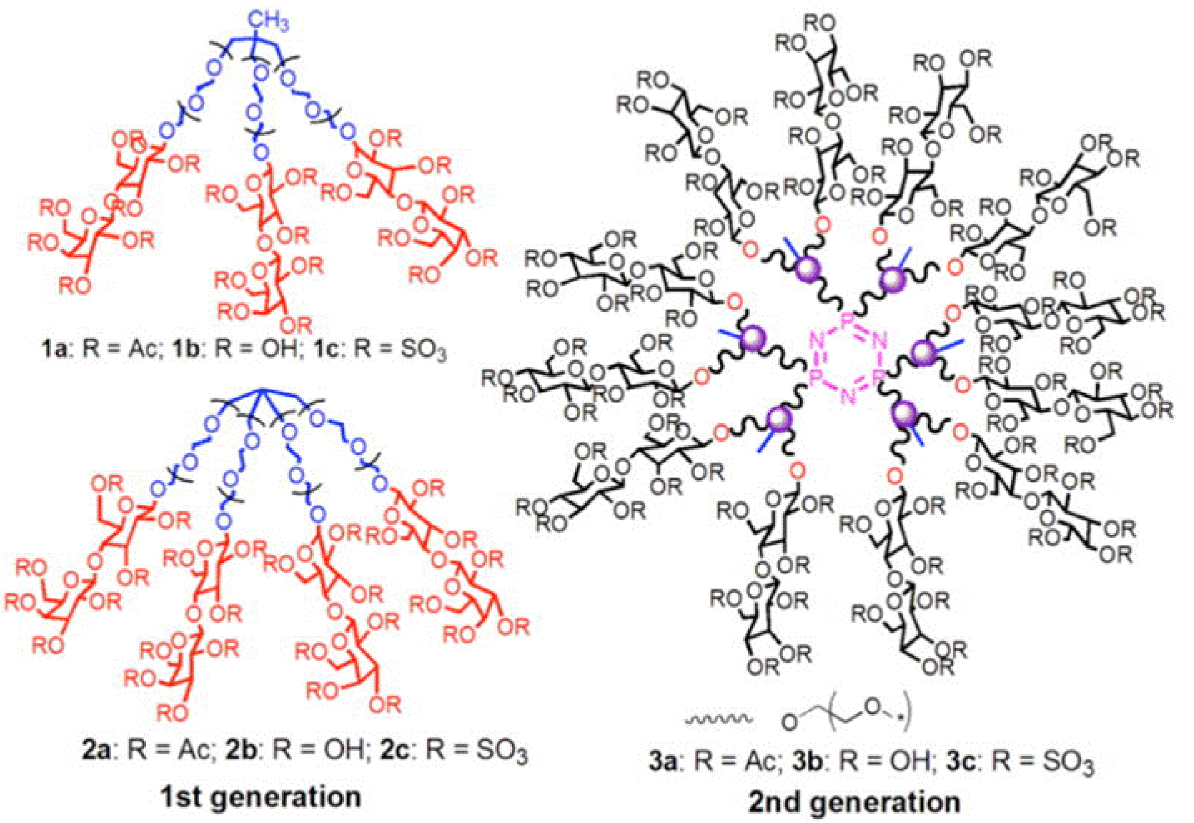

**FIGURE 5.** Structure of PEO glycosylated three-arm (1a, b, and c) and four-arm (2a, b, and c) "stars" and $N_3P_3$ core–based PEO glycodendrimers (3a, b, and c). (Reprinted with permission from Rele et al. *J. Am. Chem. Soc.* **127**, 10132–10133. Copyright 2005 American Chemical Society.)

in its binding pocket. The dendrimer did not inhibit the adhesion to P-selectin either (contrary to heparin), but selectively blocked the adhesion to L-selectin in a dose-dependent manner with an $IC_{50}$ = 2.4 nM. No explanation is given regarding the different behavior of heparin and sulfated PEO dendrimer towards P-selectin. It is also surprising that a compound acting solely through L-selectin blockade is able to block *in vivo* leukocyte extravasation. Nevertheless, applicability of these data is encouraging insofar as the sulfated PEO dendrimer has no antithrombin activity, whereas the clinical use of heparin is limited due to its anticoagulant effects.

Following the same line, Dernedde et al.[53] designed dendritic polyglycerol sulfates (dPGS) as heparin analogs (Fig. 6). To delineate structure-activity relationships, two structural features of dendrimers had been varied: the core size (MW between 2,500 and 6,000 Da) and the degree of sulfation (0 [for dPG] to 61 [for $dPGS_{61}$] sulfate groups per dendrimer), leading to the screening of six dendritic polyglycerol. In addition, a triglycerol sulfate was also included (MW = 650 Da).

In a first part of their work, the authors evaluated the binding properties of dPGS towards selectins using a surface plasmon resonance (SPR)–based binding assay. Selectin ligands were bound on a chip and E-, L-, or P-selectins functionalized gold particles. If dPGS bound to selectins, gold particles did not interact with the chip. As awaited, polyanionic dPGS did not inhibit the binding of E-selectin–coated particles. To block the interaction of L-selectin with its ligand, the most active dendrimer was dPGS61 (MW = 12,300 Da) with an $IC_{50}$ = 8 nM. The small triglycerol sulfate had an $IC_{50}$ = 2 mM. More surprisingly, the unfractionated heparin linear polymer (carrying approximately 63 sulfate groups, MW around 15,000 Da) had an $IC_{50}$ = 12 µM in the same conditions. In this assay, the comparison of the values of $IC_{50}$ of the dendrimers indicates that both the number of sulfate groups and the core size are pivotal determinants of

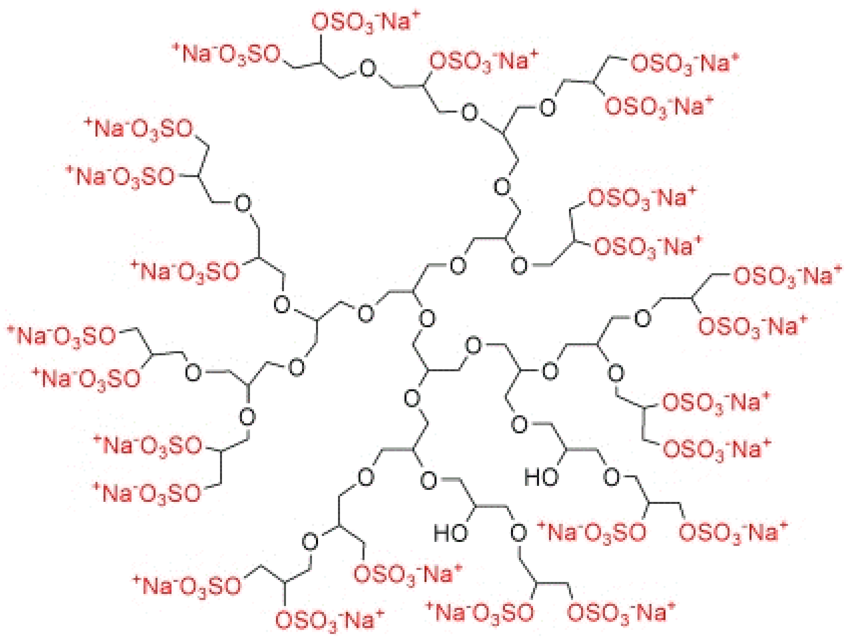

**FIGURE 6.** Representative structure of the PEO-sulfated dendrimers used in Dernedde et al.[53]. (© PNAS, reproduced with permission.)

the bioactivity. The inhibitory property of $dPGS_{61}$ was confirmed in a realistic cell-to-cell binding assay between a leukocyte cell line expressing L-selectin and HUVECs activated to express L-selectin ligand. It is noteworthy that, contrary to the data afforded in Dernedde et al.[53] with the second generation $N_3P_3$-based PEO dendrimer, dPGS have also an inhibitory activity towards P-selectin binding.

As dPGS target both L-selectin on leukocytes and P-selectin on endothelial cells, the active $dPGS_{61}$ should inhibit leukocyte extravasation to inflammatory sites. This has been proven *in vivo* in a mouse model of acute allergic contact dermatitis with typical symptoms: redness, ear swelling, edema, and cellular infiltration. To induce contact dermatitis, mice were sensitized at day 0 by a first application of trimellitic anhydride (TMA) and challenged at day 5 by a second application of TMA on ears. One hour prior to the challenge application, test compounds ($dPGS_{61}$ [at 3, 10, and 30 mg/kg], heparin, and prednisolone as a positive control [at 30 mg/kg]) were injected subcutaneously. Twenty-four hours later, ear swelling was evaluated and showed that both prednisolone and $dPGS_{61}$ clearly reduced the ear swelling in a dose-dependent manner for $dPGS_{61}$. The benefit of the tested compounds on the ear swelling can be assigned to the reduction of leukocyte extravasation to inflamed ear tissue as the enzymatic activity of neutrophil elastase was dramatically reduced in ear homogenates from mice treated with prednisolone or $dPGS_{61}$ (with a maximal effect already at the lowest dose of 3 mg/kg).

Finally, the anti-inflammatory effect of $dPGS_{61}$ also lies in the fact that it inhibits the generation of anaphylatoxin C5a (which causes enhanced vascular permeability) as shown in a mouse model of complement activation *in vivo*. SPR experiments showed that this inhibition is due to the binding of $dPGS_{61}$ on the C5 glycoprotein of the complement system, which is the precursor of C5a. This binding should occur through multivalent electrostatic interactions between the polyanionic dPGS61 and positively charged protein motifs of C5, as hypothesized for the inhibition of L- and P-selectins.

## CONCLUDING REMARKS

Among the potential applications of dendrimers in the nanomedicine landscape, the anti-inflammatory properties of dendrimers *per se* are the most recently discovered. This mini-review is based on the results presented in five main articles in which three families of dendrimers were evaluated: PAMAM-based dendrimers[33,48], phosphorus-based dendrimers of the Majoral-Caminade team[46], and PEO/PEG dendrimers[52,53]. In three out of five cases[46,52,53], the anti-inflammatory dendrimers are polyanionic nanodevices, and Dernedde et al.[53] especially afford data indicating that both the size and the polyanionic features of PEO dendrimers are crucial for their bioactivity, which appears to be based on multivalent electrostatic interactions with immune molecular partners. In the phosphorus-based dendrimer family, we have also shown that the anionic density at the surface of the dendrimer is pivotal for bioactivity[40]. Nevertheless, we also published results showing that the anionic character of the phosphorus-containing surface group is not the only critical parameter and that its precise chemical structure can be tuned to get an improved bioactivity[34,38,43].

In the work by Shaunak et al.[33], the anti-inflammatory PAMAM dendrimer bears nine glucosamine monosaccharides (dendrimer DG), whereas the same dendrimer with nine 6-O-sulfated glucosamine residues (dendrimer DGS) has no anti-inflammatory effect, but rather antiangiogenic properties. Conversely, the highly sulfated PEO glycodendrimer synthesized by Rele et al. exhibits anti-inflammatory properties[52]. Therefore, one can hypothesize that the sulfate group density of DGS is not sufficient to display anti-inflammatory activity.

Intriguingly, the most active PAMAM dendrimers revealed in Chauhan et al.[48] are amino- (G4-$NH_2$, which should be positively charged in physiological conditions) or hydroxyl-terminated (G4-OH, G4-AEEA) nanostructures. The carboxy-terminated dendrimers G4-$CO_2H$ and G4-SUC have much weaker anti-inflammatory effects.

Once anti-inflammatory properties of different dendrimers have been exemplified in various animal models of acute and chronic inflammatory disorders, and supported by complementary *in vitro* experiments, what is the future of these drug candidates? According to the “rule of 5” proposed originally in 1997[54], the potential for a chemical compound to rank among drug candidates requires no more than five H-bond donors, 10 H-bond acceptors, a molecular weight under 500 Da, and a calculated logP under 5 (it is a measure of the differential solubility of a compound between water and an immiscible solvent such as octanol). None of the anti-inflammatory dendrimers reviewed in this article fulfilled the first three “requirements”. For instance, the phosphorus-based dendrimer ABP comprises 90 oxygen and 27 nitrogen atoms (in addition with 33 phosphorus and six sulfur atoms); its molecular weight is 5820 Da. Nevertheless, a forerunner drug-candidate dendrimer has recently reached phase II clinical trials[31]. A breach has been made in the dogma that other dendrimers may use. The use of nanomaterials (including dendrimers) as drug carriers or medical imaging reagents should also benefit the development of dendrimers themselves as drugs. Indeed, the development of novel drug-delivery nanosystems such as dendrimers requires us to determine accurate regulatory issues regarding physicochemical characterization (PCC) and absorption, distribution, metabolism, and excretion (ADME) studies[55]. Pharmacokinetic and toxicology aspects also play an important role in the design and clinical development of dendrimers as nanocarriers and imaging reagents; in this respect, much more data are available[56].

Another consideration that promises the advent of new therapeutics is the crucial need of innovative therapy for the treatment of chronic inflammatory diseases (CIDs). CIDs are medical conditions characterized by persistent inflammation due to an inappropriate, uncontrolled response of the immune system to either endogen (autoimmune) or exogen stimuli (environmental factors). People with CIDs tend to undergo a great deal of suffering and disabling disadvantages. CIDs include, among others, rheumatoid arthritis, psoriasis, inflammatory bowel diseases (such as ulcerative colitis and Crohn’s disease), atherosclerosis, chronic obstructive pulmonary disease (COPD), and neurodegenerative CIDs such as multiple sclerosis and Alzheimer’s disease. Given the pivotal role of proinflammatory (upstream in the inflammatory cascade) and inflammatory (downstream in the inflammatory cascade) cytokines in the onset, the development, and the persistence of CIDs, tremendous efforts have been made to chart the cytokine network in each CID. This has led to the development of biological therapeutics: monoclonal antibodies (directed against these cytokines or their receptors) or soluble receptors neutralizing these cytokines to treat CID. Although these biotherapies have been highly effective, they also have strong drawbacks essentially regarding secondary risks (increase of infections and malignancies) and cost (between US$10,000 and 15,000/patient/year for long-term treatments that only suspend the disease without curing it). When taking into account all these elements, they exacerbate the urgent problem raised in developed societies with regard to aging and health care costs, in particular in relation to Alzheimer's disease, whose prevalence is scheduled to strongly increase in the coming decades. This is one of the reasons why the *NewYork Times* is writing so much about Alzheimer's disease therapies[57].

## ACKNOWLEDGMENTS

We thank Ms. Amy Cross for English editing of the manuscript.
